# Earthquake-related versus non-earthquake-related injuries in spinal injury patients: differentiation with multidetector computed tomography

**DOI:** 10.1186/cc9391

**Published:** 2010-12-29

**Authors:** Zhi-hui Dong, Zhi-gang Yang, Tian-wu Chen, Zhi-gang Chu, Qi-ling Wang, Wen Deng, Joseph C Denor

**Affiliations:** 1 Department of Radiology, West China Hospital of Sichuan University, Chengdu 610041, China; 2 State Key Laboratory of Biotherapy, West China Hospital of Sichuan University, Chengdu 610041, China; 3 Department of Senior Services, Methodist Hospital, Park Nicollet Clinic, St. Louis Park, MN 55416, USA

## Abstract

**Introduction:**

In recent years, several massive earthquakes have occurred across the globe. Multidetector computed tomography (MDCT) is reliable in detecting spinal injuries. The purpose of this study was to compare the features of spinal injuries resulting from the Sichuan earthquake with those of non-earthquake-related spinal trauma using MDCT.

**Methods:**

Features of spinal injuries of 223 Sichuan earthquake-exposed patients and 223 non-earthquake-related spinal injury patients were retrospectively compared using MDCT. The date of non-earthquake-related spinal injury patients was collected from 1 May 2009 to 22 July 2009 to avoid the confounding effects of seasonal activity and clothing. We focused on anatomic sites, injury types and neurologic deficits related to spinal injuries. Major injuries were classified according to the grid 3-3-3 scheme of the Magerl (AO) classification system.

**Results:**

A total of 185 patients (82.96%) in the earthquake-exposed cohort experienced crush injuries. In the earthquake and control groups, 65 and 92 patients, respectively, had neurologic deficits. The anatomic distribution of these two cohorts was significantly different (*P *< 0.001). Cervical spinal injuries were more common in the control group (risk ratio (RR) = 2.12, *P *< 0.001), whereas lumbar spinal injuries were more common in the earthquake-related spinal injuries group (277 of 501 injured vertebrae; 55.29%). The major types of injuries were significantly different between these cohorts (*P *= 0.002). Magerl AO type A lesions composed most of the lesions seen in both of these cohorts. Type B lesions were more frequently seen in earthquake-related spinal injuries (RR = 1.27), while we observed type C lesions more frequently in subjects with non-earthquake-related spinal injuries (RR = 1.98, *P *= 0.0029).

**Conclusions:**

Spinal injuries sustained in the Sichuan earthquake were located mainly in the lumbar spine, with a peak prevalence of type A lesions and a high occurrence of neurologic deficits. The anatomic distribution and type of spinal injuries that varied between earthquake-related and non-earthquake-related spinal injury groups were perhaps due to the different mechanism of injury.

## Introduction

The magnitude 8.0 Sichuan earthquake that happened at 2:28 PM Beijing time on 12 May 2008 injured an estimated 374,643 people. A retrospective study of the features of 223 patients with spinal injuries was performed using multidetector computed tomography (MDCT). This study relied on the 2,728 patients with earthquake-related spinal injuries seen in a key university hospital [1]. Located 92 km from the Wenchuan County epicenter, this 4,300-bed hospital served as an important intact rescue center immediately following the earthquake.

Spinal injuries account for 13.0-15.2% of earthquake-related trauma patients [2,3] and 1.6-10% of all other trauma patients [4,5]. In general, the major etiologies of spinal injuries include motor vehicle accidents, falls, athletic mishaps, penetrating injuries and industrial accidents [6,7]. In contrast, the spinal injuries sustained by the 223 patients during the Sichuan earthquake usually occurred from crush injuries followed by falls. Although the features of earthquake-related spinal injuries have been studied [1], no one has reported the differences between earthquake-related and other spinal injuries. We propose that there is a difference between these two types of injuries on the basis of their MDCT features because of their different injury mechanisms. MDCT is a fast and reliable modality to determine the pattern and severity of spinal injuries and the degree of spinal instability [8].

Classification based on the information provided by MDCT should provide concise information regarding the severity of the injury and guide the choice of treatment. We used MDCT alone to evaluate the difference between spinal injuries from earthquake victims and those from other causes, which may help in clinical management algorithms. We cite better disaster preparedness as an overarching goal of this investigation.

## Materials and methods

### Patients

The study was approved by the ethics committee of our medical school, and because of the retrospective nature of this study, informed consent was waived. Of the 2,728 patients with earthquake-related spinal injuries seen in our hospital, we consecutively enrolled patients into the exposed cohort according to the following criteria: (1) the etiology of the injuries was associated with the 2008 Sichuan earthquake, (2) spinal injuries were evaluated using MDCT, and (3) the patients had not received related surgical treatment before the spinal MDCT scan. To avoid bias, we excluded patients injured in an earthquake-related motor vehicle accident. With the exception that the etiology of spinal injury was not associated with the earthquake, similar inclusive criteria were used to enroll patients into the cohort of patients with other common injuries (unexposed cohort). By 4 June 2008, we had enrolled all 223 patients with clinically worrisome spinal injuries from the Sichuan earthquake who met the inclusive criteria. We selected an unexposed cohort similar in size to the exposed cohort of 223 consecutive patients. To avoid the confounding effect of seasonal activity and clothing, our unexposed cohort was collected during a similar time frame of 1 May 2009 to 22 July 2009.

### MDCT protocols

Among the earthquake-exposed cohort, spinal CT scans of 10, 175 and 38 patients were performed using the Philips Brilliance 64-slice MDCT (Philips Healthcare, Eindhoven, the Netherlands), the Siemens Somatom Sensation 16-slice MDCT and the Siemens Somatom Plus 4-slice MDCT (Siemens Medical Systems, Forchheim, Germany), respectively. Among the unexposed common injury cohort, the 223 spinal CT scans were performed using the Siemens Somatom Sensation 16-slice MDCT. Each patient underwent MDCT along the *z*-axis from the upper two vertebrae to the lower two vertebrae of the target vertebrae. Scanning parameters were 140 kV, 250 mAs, rotation time 1.0 s, pitch 1.235 and collimation of 64 × 0.625 mm for the Philips Brilliance 64 MDCT scanner; 120 kV, 250 mAs, rotation time 0.75 s, pitch 1.0 and collimation of 16 × 0.75 mm for the Siemens Somatom Sensation 16-MDCT scan; and 120 kV, 180 mAs, rotation time 0.75 s, pitch 1.5 and collimation of 4 × 1.0 mm for the Siemens Somatom Plus 4 MDCT scan. The axial image data were reconstructed in 1- to 2-mm thickness and 0.7- to 1.0-mm increments and transferred to syngo Workflow software of the picture achieving and communicating system work station (PACS; Siemens Medical Solutions).

### Image analysis

All MDCT scans were read independently by two experienced radiologists (ZGY and ZHD, with 23 and 9 years of experience, respectively) on a syngo Workflow PACS workstation on which we obtained axial images and multiplanar reformatted sagittal and coronal images. Surface-shaded display was used to evaluate the vertebral fractures and the deformation of the spinal column. Discrepancies in interpretation were resolved by consensus.

At each vertebral site, the presence of fractures in the vertebral body, including the transverse processes, spinous processes, articular processes and isthmus of the vertebrae, as well as the degree of spinal canal narrowing, were evaluated, with differences resolved by consensus. We categorized the injury patterns and regions of spinal injuries and correlated these findings with the mechanism of injury and the clinician-evaluated Frankel level [6]. Spinal injuries were subdivided into major and minor injuries [9].

**Figure 1:**
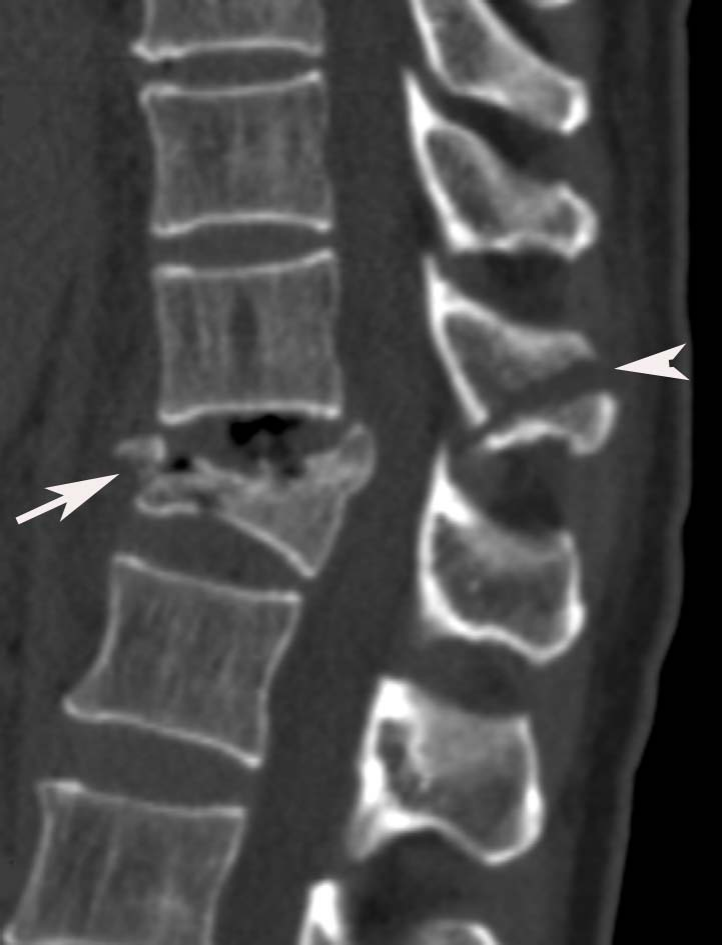
**Reformatted sagittal image clearly shows spinous process distraction of T11 (arrowhead) and associated burst fracture (type A3) of T12 (arrow)**. Thus this is a Magerl (AO: Arbeitsgemeinschaft fur Osteosynthesefragen classification) type B2 lesion (posterior disruptions that predominantly involved osseous).

**Figure 2:**
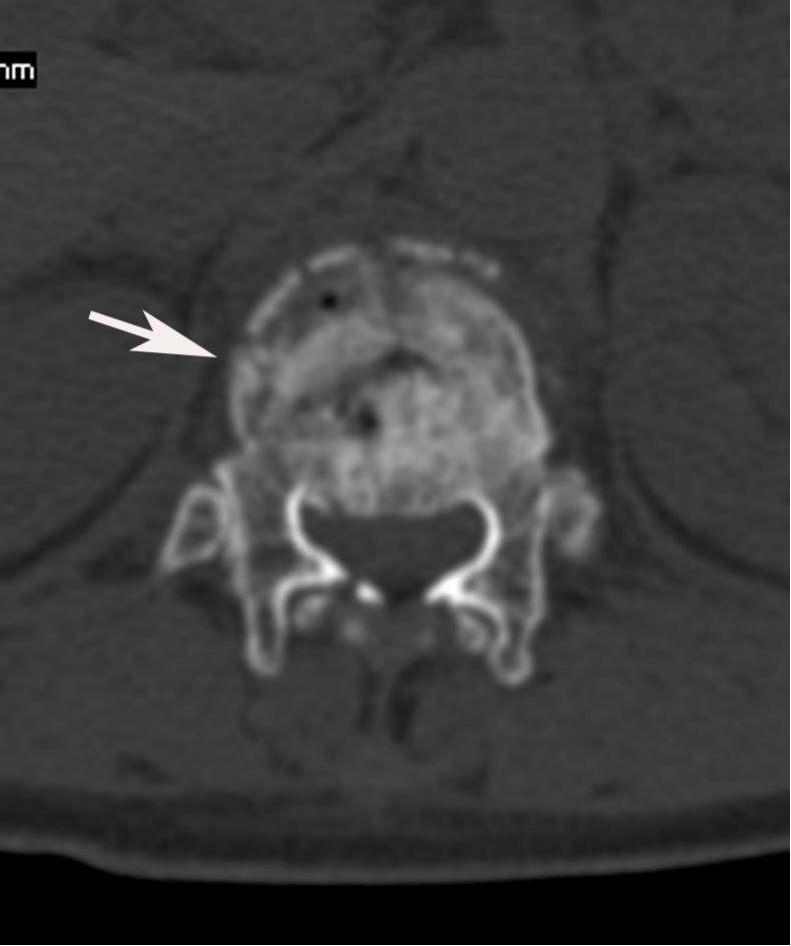
**Cross-sectional image of the same patient as in Figure 1 clearly shows the associated burst fracture (type A3) of T12 (arrow) and the spinal canal narrowing degree of 1 (constriction of one third of the spinal canal)**.

**Figure 3:**
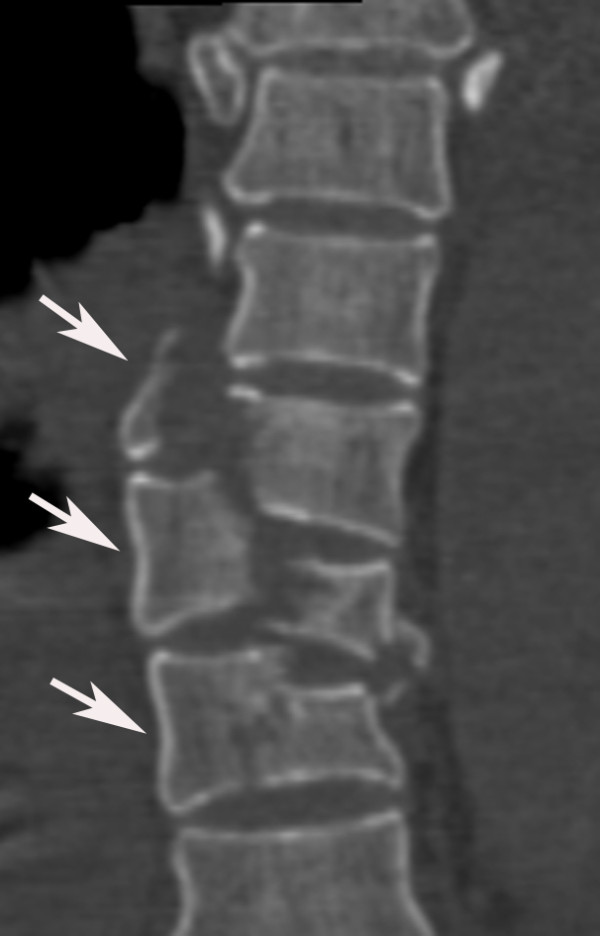
**Reformatted coronal image of a 51-year-old female patient who had crush trauma as a result of an earthquake shows an oblique fracture from T5 through T7 and form type C3 lesions**.

**Figure 4:**
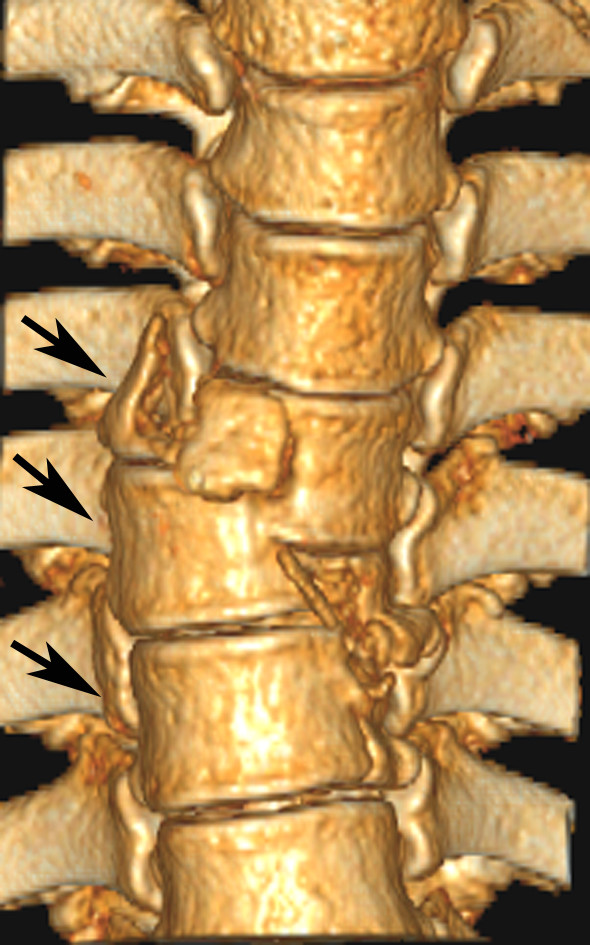
**Surface-shaded display of a 51-year-old female patient shows type C3 lesions from T5 through T7**.

Major spinal injuries were categorized according to the Magerl (AO) classification because it allows categorization of injuries to all relevant parts of the spine (Figures 1, 2, 3, 4) [10-12]. As for Magerl AO types, spinal injuries are grouped as type A, type B and type C lesions. Type A (compression injuries of the anterior column) is subdivided into A1 (impaction fractures), A2 (split fractures) and A3 (burst fractures). Type B (distraction injuries of the anterior and posterior columns with transverse disruption) is subdivided into B1 (posterior disruptions that are predominantly ligamentous), B2 (posterior disruptions that are predominantly osseous) and B3 (anterior disruptions through the disk). Type C (anterior and posterior element injuries with superimposed rotation resulting from axial torque) is subdivided into C1 (type A injuries with rotation), C2 (type B injuries with rotation) and C3 (rotational shear injuries). Injuries of three to seven cervical vertebrae were categorized according to the Magerl AO classification because they were similar to injuries of the thoracic and lumbar spine. We classified teardrop fractures, occipital condyle fractures, Jefferson fractures, odontoid fractures, hangman's fractures, lateral mass fractures and posterior arch or laminar fractures as major injuries. Minor injuries, on the other hand, included isolated or combined fractures of transverse processes, facets, pars interarticularis, spinous process and atlantoaxial subluxation.

Spinal canal narrowing was measured in all injured vertebrae at the point of greatest narrowing and was defined as 0 (no constriction of the spinal canal), 1 (constriction of one third of the spinal canal), 2 (constriction of two thirds of the spinal canal) and 3 (constriction of all of the spinal canal), respectively [13]. The neurologic function impairment grades were classified according to the widely accepted Frankel grading method because of its ease of use and lack of subjectivity [6]. Patients were graded as Frankel level of (A) complete, (B) sensory only, (C) motor useless, (D) motor useful and (E) recovery.

### Statistical analysis

Data for each patient were entered into a Microsoft Excel database (Microsoft Corporation, Redmond, WA, USA). We performed data analysis on a personal computer using the SPSS statistical package (version 13.0 for Windows; SPSS Inc., Chicago, IL, USA). We determined the differences between the earthquake-exposed and non-earthquake-exposed cohorts using risk ratios (RRs) and the Mann-Whitney *U *test for gender, age (categorized as ages <35 years, 35-64 years and >64 years) and anatomic distribution of injury (categorized as cervical, thoracic and lumbar spine). Additionally, the anatomic distributions and types of injuries were compared using the χ^2 ^test, and the patient's age and spinal canal narrowing degrees were compared using the Mann-Whitney *U *test. The Kruskal-Wallis H test was performed to compare the spinal canal narrowing degrees between different AO types of injuries. We accepted two-tailed *P *values less than 0.05 as a statistically significant difference.

## Results

The earthquake-exposed cohort included 119 (53.36%) female patients and 104 (46.64%) male patients, with ages ranging from 3 to 89 years (mean age, 45 years) including 56, 118 and 49 patients grouped by ages <35, 35-64 and >64 years, respectively. The mean hospital stay was 13.6 days, ranging from 1-203 days. Only one 74-year-old female patient died as a result of heart failure from septic shock related to thoracoabdominal blunt trauma. The non-earthquake-related cohort included 53 (23.77%) female patients and 170 (76.23%) male patients whose ages ranged from 1.8 to 88 years (mean age, 40 years) including 80, 124 and 19 patients in the age groups <35, 35-64 and >64 years, respectively. The mean hospital stay was 11 days and ranged from 1-113 days. Three patients died, including one patient whose spinal injury was combined with a head injury, one patient with an injury of cervical spine due to a gunshot wound and one patient with multiorgan injury. Male patients were statistically more common in the non-earthquake-related spinal injury cohort (RR = 1.63, Mann-Whitney *U *test = 6.418 and *P *< 0.001). We also found that young patients more commonly represented in the non-earthquake-related injury cohort (*Z*-score = -3.753, *P *< 0.001).

### Cause and anatomical distribution of spinal injuries

**Figure 5:**
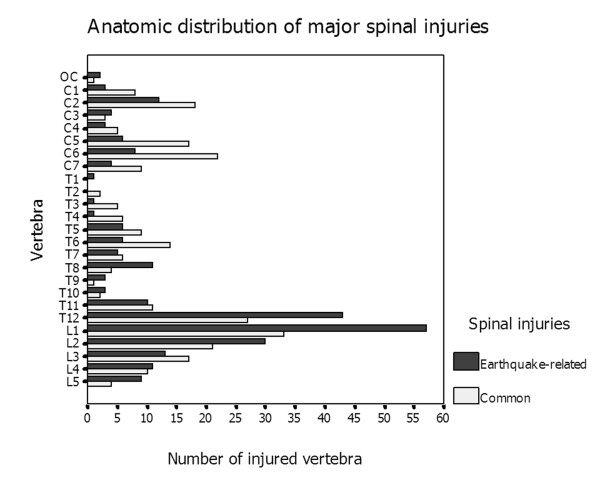
**Clustered bar chart shows that earthquake-related major spinal injuries were most commonly seen in the lumbar spine with the peak prevalence being in the T12-L2 vertebra**. Non-earthquake-related major spinal injuries were distributed equally in the cervical, thoracic, and lumbar spine.

**Figure 6:**
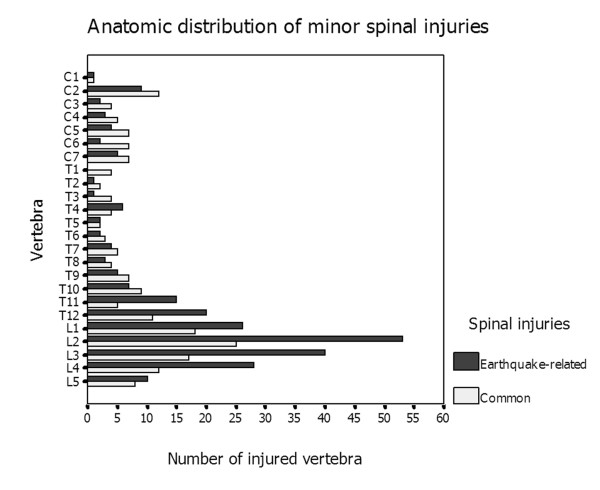
**Clustered bar chart shows that minor earthquake-related spinal injuries were more frequently involved in the lumbar spine than non-earthquake-related injuries, with the peak incidence in the T12-L4 vertebra**.

Of the earthquake-exposed cohort, 185 patients (82.96%) sustained crush injuries and 38 had fallen with resultant injury. For the non-earthquake-related spinal injury cohort, on the other hand, the proximate causes of injury included falls (90 patients), motor vehicle accidents (71 patients), crush injuries (26 patients), assault and gunshot wounds. The numbers and anatomic distribution of spinal injuries detected in the earthquake-exposed and non-earthquake-exposed cohorts are listed in Table 1. We found a significant difference in the anatomic distribution of these two cohorts (χ^2 ^= 19.457, *P *< 0.001). The cervical spinal injuries presented more frequently in patients with non-earthquake-related spinal injuries than in patients with earthquake-related injuries (RR = 2.12, Mann-Whitney *U *test = 5.739 and *P *< 0.001), including both the cervical major spinal injuries (RR = 1.95, Mann-Whitney *U *test = 4.148 and *P *< 0.001) (Figure 5) and the cervical minor spinal injuries (RR = 2.25, Mann-Whitney *U *test = 3.661 and *P *< 0.001) (Figure 6). Multilevel major injuries were detected in 59 patients (34.10% of 173 patients) in the earthquake-exposed cohort (two to five vertebrae; average, two vertebrae), of which 22 patients (37.29%) did not have an adjacent spinal level injury. Similarly, 61 patients (39.35% of 155 patients) had multilevel involvement in the non-earthquake-related spinal injury cohort (two to six vertebrae; average, two vertebrae), with 26 patients (42.62%) not presenting with adjacent vertebral involvement.

**Table 1:** Number and anatomic distributions of spinal injuries detected in earthquake-related and non-earthquake-related injuries

Injury	Earthquake-related injuries (exposed cohort)	Non-earthquake-related injuries (unexposed cohort)
Type of injury	Number of patients/vertebrae	
Spinal injuries	198/501	167/438
Major injuries	173/252	155/255
Minor injuries	119/249	94/183
Anatomic distributions (vertebrae)	Major/minor injuries	Major/minor injuries
Cervical spine	42/26	83/43
Thoracic spine	90/66	87/60
Lumbar spine	120/157	85/80

### Types of spinal injuries

**Figure 7:**
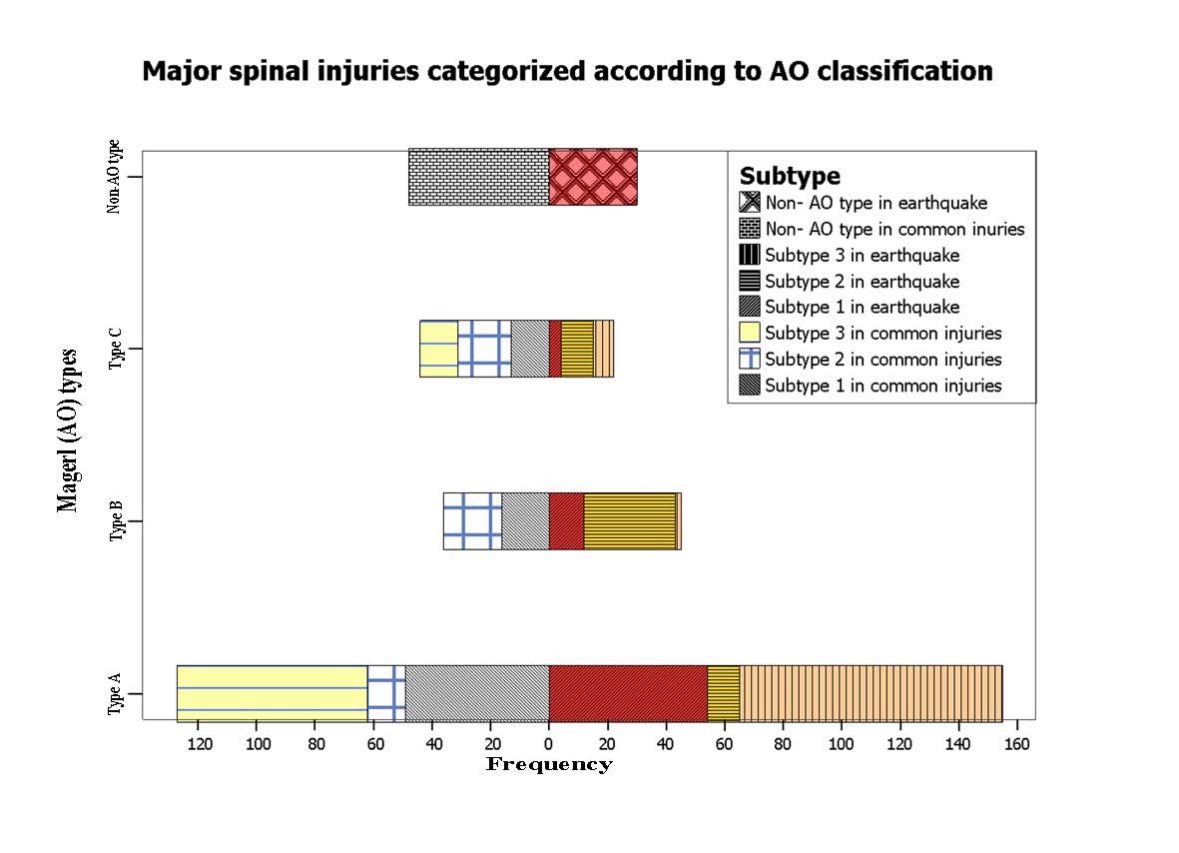
**Stacked bar chart shows that type A spinal injuries were the main type of AO spinal injuries detected in both earthquake-related and non-earthquake-related spinal injuries**. In earthquake-related spinal injuries, type A3 injuries compose 58.06% of type A injuries.

Of the 252 earthquake-related major spinal injuries, types A, B and C lesions were detected in 155, 45 and 22 vertebrae, respectively, which was significantly different from the findings in the non-earthquake-related cohort, in whom types A, B and C lesions were detected in 127, 36 and 44 vertebrae, respectively (Figure 7) (χ^2 ^= 15.250, *P *= 0.002). Type C lesions were seen more commonly in the non-earthquake-related spinal injury patients than in the earthquake-related spinal injury patients (RR = 1.98, Mann-Whitney *U *test = 2.760 and *P *= 0.0029). However, the incidence of type B lesions was relatively higher in patients with earthquake-related injuries (20.27% of AO type fractures) than in those with non-earthquake-related spinal injuries (RR = 1.27). Associated type A3 fractures were detected in 31 earthquake-related type B lesions (72.09% of the 43 type B lesions that associate with type A fractures). Other categories of major injuries are listed in Table 2. Of the minor injuries, transverse process fractures were the main type of injury in both cohorts (Table 3).

**Table 2:** Non-AO-type classification of major spinal injuries^a^

Types of fractures	Earthquake-related vertebral injuries (%)	Non-earthquake-related vertebral injuries (%)
Teardrop	4 (13.33)	13 (27.08)
Occipital condyle	2 (6.67)	1 (2.08)
Jefferson fractures	1 (3.33)	5 (10.42)
Odontoid processes	5 (16.67)	10 (20.83)
Hangman's fractures	4 (13.33)	8 (16.67)
Lateral mass or anterior arch	4 (13.33)	3 (6.25)
Posterior arch or laminar fractures	10 (33.33)	8 (16.67)
Total	30 (100)	48 (100)

^a^Non-AO-type classification represents classifications of major spinal injuries that cannot be classified by the grid 3-3-3 scheme of the AO fracture classification established by Magerl [6,10].

**Table 3:** Minor injuries detected in earthquake-related and non-earthquake-related injuries

Injuries	Earthquake-related vertebral injuries (%)	Non-earthquake-related vertebral injuries (%)
Transverse process fractures	155 (62.25)	112 (61.20)
Spinous process fractures	36 (14.46)	29 (15.85)
Facet fractures	16 (6.42)	4 (2.19)
Combined fractures	33 (13.25)	28 (15.30)
Atlantoaxial subluxation	9 (3.61)	10 (5.46)
Total	249 (100)	183 (100)

### Spinal canal narrowing and neurologic deficit

The degree of spinal canal narrowing in both cohorts is listed in Table 4. No statistically significant difference was found in these two cohorts (*Z*-score = -0.727, *P *= 0.467). The degree of spinal canal narrowing increased significantly from Magerl type A through type C injuries (χ^2 ^= 14.4, *P *= 0.001, for earthquake-exposed patients; χ^2 ^= 47.023, *P *< 0.001, for the non-earthquake-exposed cohort) (Table 4).

**Table 4:** Degrees of major injuries involving spinal canal narrowing^a^

	Spinal canal narrowing degrees (vertebrae)				
Cohort	0	1	2	3	Total
Earthquake-related injuries	110	76	50	16	252
Non-earthquake-related injuries	129	56	44	26	255
AO type	Earthquake-related/non-earthquake-related (vertebrae)				
Type A	68/72	47/29	34/22	6/4	155/127
Type B	11/16	20/7	10/8	4/5	45/36
Type C	3/2	7/13	6/14	6/15	22/44

^a^χ^2 ^= 14.4, *P *= 0.001, for earthquake-related injuries. χ^2 ^= 47.023, *P *< 0.001, for non-earthquake-related injuries.

Neurologic deficits such as decreased perineal sensation, limb anesthesia, bladder dysfunction, muscle weakness and paraplegia occurred in 65 patients (29.15%; 95% CI, 23.19-35.11%) in the earthquake-related cohort, with a Frankel level of A, B, C and D in 14, 8, 24 and 19 patients, respectively. For the non-earthquake-related cohort, neurologic deficits occurred in 92 patients (41.26%; 95% CI, 34.80-47.72%), with a Frankel level of A, B, C and D in 41, 12, 24 and 15 patients, respectively. Spinal canal narrowing was much higher in patients with neurologic deficits than in patients without neurologic deficits (Table 5) (*Z*-score = -3.498 for earthquake-exposed cohort and *Z*-score = -10.382 non-earthquake-exposed cohort, respectively; *P *< 0.001). Furthermore, the incidence of neurological deficit increased significantly from Magerl type A through type C: neurological deficit was associated with 21.99% of type A lesions, 40.74% of type B lesions and 69.70% of type C lesions (χ^2 ^= 57.156, *P *< 0.001).

**Table 5:** Degrees of spinal canal narrowing in patients with or without neurologic deficit^a^

	Narrowing degrees (patients)				
Neurologic deficit	0	1	2	3	Total
Earthquake-related injuries					
Number of patients (with/without)	18/63	15/61	21/29	11/5	65/158
Non-earthquake-related injuries					
Number of patients (with/without)	12/104	24/20	33/6	23/1	92/131

^a^*Z*-score = -3.498, *P *< 0.001 for earthquake-related cohort. *Z*-score = -10.382, *P *< 0.001 for non-earthquake-related spinal injuries.

## Discussion

Spinal injuries are common in both trauma associated with earthquakes and other major blunt traumas [2-5]. A spinal cord injury can be disabling or life threatening with poor long-term physical and psychological consequences [14-16]. Non-earthquake-related spinal injuries rarely occur as a result of crush injury [8,17]. In our earthquake-exposed cohort, however, crush injury was the leading cause of spinal injury. As spinal injuries occur most commonly in males during the summer months and on weekends [6], we chose a non-earthquake-related cohort from a similar time of the year in 2009 to avoid the bias introduced by the confounding effect of seasonal activity and clothing.

We found young male patients were more commonly involved in non-earthquake-related spinal injuries, which concurs with previous data. This difference between the cohort populations can probably be attributed to the fact that falls and motor vehicle injuries are the two primary causes of non-earthquake-related spinal injuries. Most work-related falls from elevated height occur in young males, and drivers injured in motor vehicle collisions also tend to be young males, consistent with previous Chinese data. In the earthquake, however, agile young males would be more likely to escape danger and withstand vertebral trauma. On the other hand, as the earthquake occurred during working hours in the mountainous area of China, most of the males were working and alert. In contrast, unemployed and older adult females were at home and relatively relaxed. Thus, the sudden, intense earthquake would be more likely to injure the latter group. Our results indicate that it was necessary to increase attention to female and older adult patients in the ward preparation and staff scheduling.

As described by Velez and Newell [6], the majority of spinal injuries in general blunt trauma occur at the level of the cervical spine, followed by thoracic, thoracolumbar and lumbosacral spinal injuries. In our study, the anatomic distributions of these two cohorts were significantly different. In our earthquake-related cohort, we found lumbar spinal injuries were most common, with the peak incidence being in the T12 to L3 region, similar to the results of a study of spinal cord injuries sustained in a Pakistani earthquake [18]. This finding possibly results from random distribution of the crush-related forces exerted on the spinal column. The larger vertebrae occupy a longer section of the spinal column, which relates to a higher chance of exposure to spinal crush injuries. In the non-earthquake-related cohort, however, both major and minor cervical spine injuries occurred with increased frequency; thus major injuries were distributed fairly equally among the cervical, thoracic and lumbar spine. Possible explanations for this finding include the cervical spine's mobility and flexibility, lack of protection by a rib cage and fragility compared to the lumbar spine. Furthermore, all traumas to the cervical spine result in a high incidence of some kind of neurologic injury-related mortality [6], which made the patients less likely to survive the disaster. We noticed that multiple fractures were common in both cohorts of this series and that more than one third of spinal injuries were not adjacent to each other, which stresses the importance of evaluating the entire spine after injury in either earthquake-related or non-earthquake-related injuries.

The mechanisms of spinal fractures include simple flexion, flexion-distraction, flexion and compression, extension, "shearing" forces and rotation [19]. Of these, compression, distraction and rotation (axial torque) are the most important mechanisms. Using mechanism of injury, pathomorphological uniformity and healing potential, Magerl established the grid 3-3-3 scheme of the AO fracture classification [10]. In a series of traumatic spinal injuries investigated by Magerl *et al*. [10], type A fracture occurred most commonly (66.1%), whereas types C and B occurred in 19.4% and 14.5% of patients, respectively. In our investigation, type A injuries composed 69.82% of AO type fractures in patients with earthquake-related injuries, with a RR of 1.23 compared to patients with non-earthquake-related injuries. The higher incidence of type A fractures was possibly a result of the direct vertical force or axial loading caused by falling objects that caused compression fracture. Types B and C injuries tend to be caused by flexion violence, which is more common in motor vehicle collisions and falls [19,20]. The comparatively high incidence of type C lesions found in the non-earthquake-related cohort of this study is consistent with these mechanisms of fall and motor vehicle trauma.

According to the AO classification system applied in the present study, earthquake-related type B lesions occurred more often than other types, with a RR of 1.27 as compared to common spinal injuries. We hypothesized that the forces of falling objects on the victims who were most likely in anterior flexion, combined with the axial loading or vertical force, might invoke a flexion and distraction component. The axis of rotation adjacent to the posterior vertebral body cortex may result in associated anterior compression. When the axis of rotation is beyond the posterior longitudinal ligament, combined type A3 compression fractures tended to occur. In this manner, all three sections of the spinal column can fail because of flexion and distraction with initial axial loading, resulting in an unstable type B lesion. The high incidence of associated type A3 fractures suggests that spinal crush injuries are different from seat belt or chance fractures, in which the initial force is flexion and distraction and the fulcrum is mainly the anterior column [7]. These results indicate that we should pay more attention to the detection and management of type B fractures in earthquake victims.

Clinically, neurologic sequelae serve as one of the most important factors in assessing spinal injuries and clinical management [4,6,9,15,16]. Of all the spinal injuries, spinal cord injuries may occur in 4.9-9.0% of patients in the early postfracture period [5-21]. In our series, the incidence of neurologic deficit was much higher in both cohorts. The incidence of neurological deficits increased significantly between AO types, which is similar to the finding of Magerl *et al*. [10]. Our study also revealed that the degree of spinal canal narrowing was higher in patients with neurologic deficits.

Minor injuries compose about 16.95% of all spinal fractures [19]. The clinical importance of these injuries is still controversial [22,23]. As shown in our study, almost half of injuries from the earthquake-related spinal injury group were minor injuries. This might be a result of the high sensitivity of MDCT in detecting these injuries. Otherwise, a wide anatomic distribution of minor fractures might reveal that the injuries in the earthquake-related injury cohort were widespread multiregional injuries, which once again stresses the need to scan the entire spinal column.

Our study has limitations. Since our hospital is a major medical center in the southwest region of China, some severely injured patients were transferred from the local hospital and may have had progression to neurologic sequelae in the process of being transported to our site. Although we selected the data consecutively to try our best to avoid selection bias, the comparatively high incidence of neurologic deficits in the non-earthquake-related cohort perhaps is due to this type of bias.

## Conclusions

Spinal injuries sustained in the Sichuan earthquake were mainly caused by crush injuries and included a high incidence of neurologic deficits. The thoracolumbar region is the most common site of spinal fracture, and type A fractures are the most common type of major injury. The major injuries of patients with non-earthquake-related spinal injuries were equally distributed among the cervical, thoracic and lumbar spine, with a comparatively high incidence of type C lesions, which were significantly different from the distribution observed among patients with earthquake-related injuries, which perhaps were due to the special mechanism associated with the earthquake.

## Key messages

• The leading cause of spinal injuries in the Sichuan earthquake was crush injuries.

• Earthquake-related spinal injuries were mainly involved in the thoracolumbar region and resulted in type A fractures with a high incidence of neurologic deficits.

• The causes of non-earthquake-related spinal injuries were significantly different from earthquake-related injuries, including falls, motor vehicle accidents, crush injuries, assaults and gunshot wounds.

• Non-earthquake-related spinal injuries were equally distributed among the cervical, thoracic and lumbar spine, with a comparatively high incidence of type C lesions.

## Abbreviations

AO: Arbeitsgemeinschaft fur Osteosynthesefragen; CI: confidence interval; CT: computed tomography; MDCT: multidetector computed tomography; PACS: picture achieving and communicating system; RR: risk ratio.

## Competing interests

The authors declare that they have no competing interests.

## Authors' contributions

ZGY and ZHD conceived and organized the study. ZHD, TWC, ZGC, QLW and WD participated in the acquisition of data. ZGY, ZHD, TWC, ZGC and JCD performed the statistical analysis and interpretation of data. ZGY, ZHD, TWC, ZGC and JCD participated in the drafting of the manuscript. All authors read and approved the final version of the manuscript.
